# Supplementary material for: Dietary cholesterol promotes steatohepatitis related hepatocellular carcinoma through dysregulated metabolism and calcium signaling
Source: Nat Commun. 2018 Oct 26;9:4490. doi: 10.1038/s41467-018-06931-6 (PMC6203711; doi:10.1038/s41467-018-06931-6)
Supplement: Supplementary file 1 — Supplementary Information [file 41467_2018_6931_MOESM1_ESM.pdf]

Supplementary Figures and Tables

Supplementary Fig. 1

HFHC (lung metastasis)

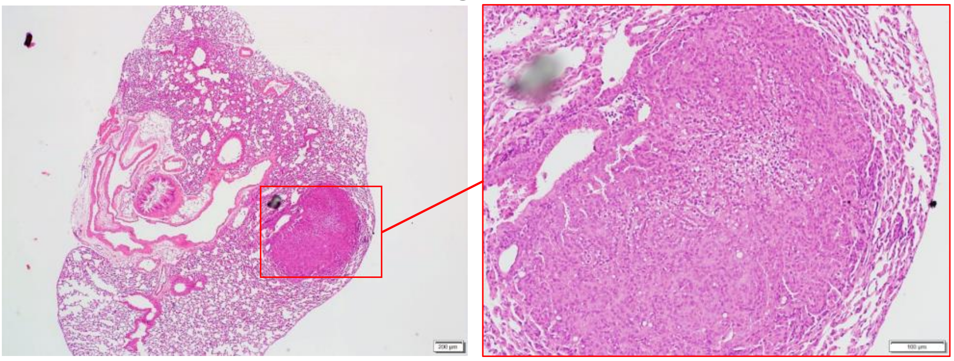

Supplementary Fig. 1. Representative H&E staining histological images of lung metastasis from a HFHC-fed mouse.

Supplementary Fig. 2

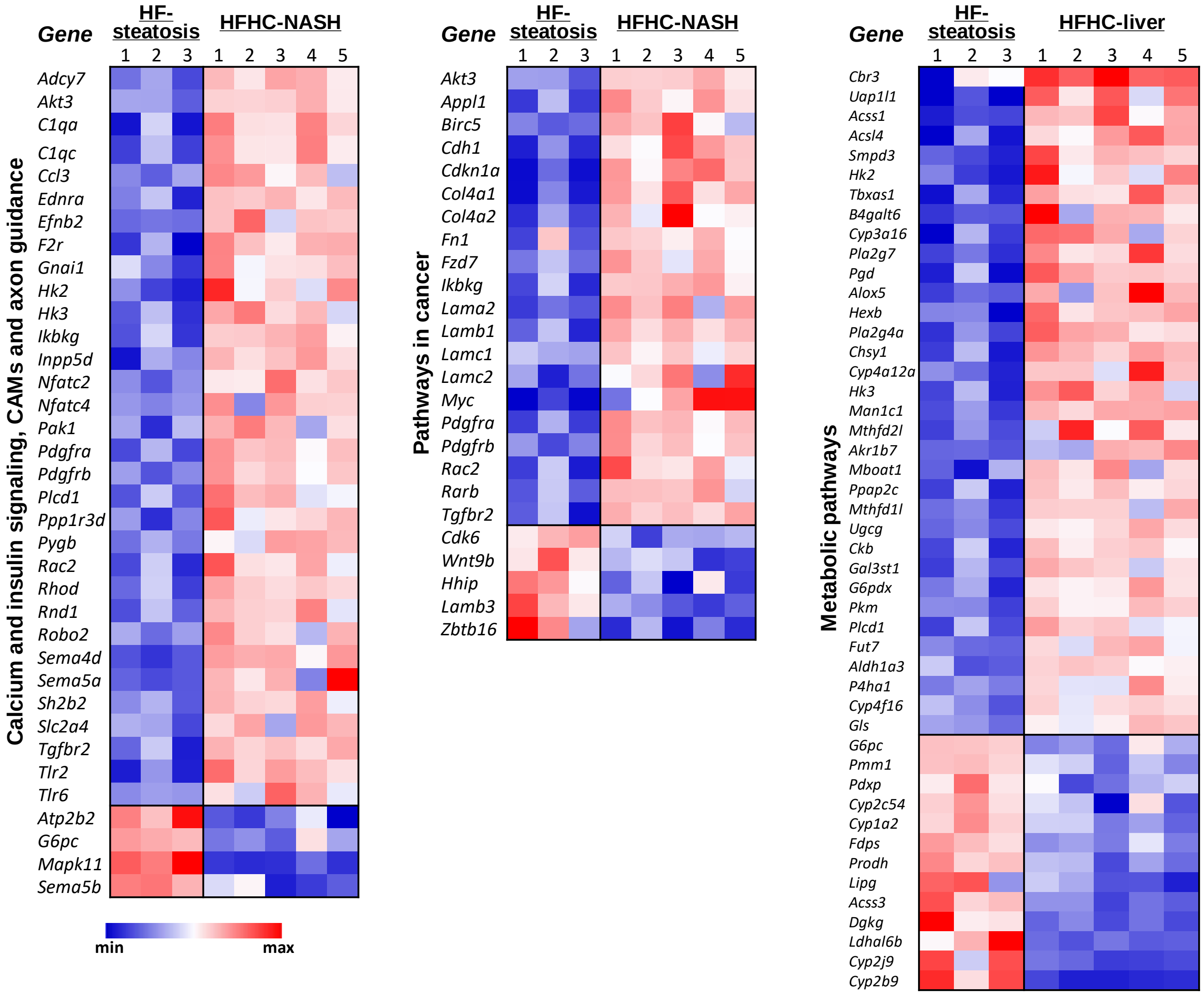

Supplementary Fig. 2 Expressional aberration of cancer- and metabolism-related genes associated with NASH development in HFHC-fed livers. Expression levels were normalized to the mean level of each gene among all samples shown.

Supplementary Fig. 3

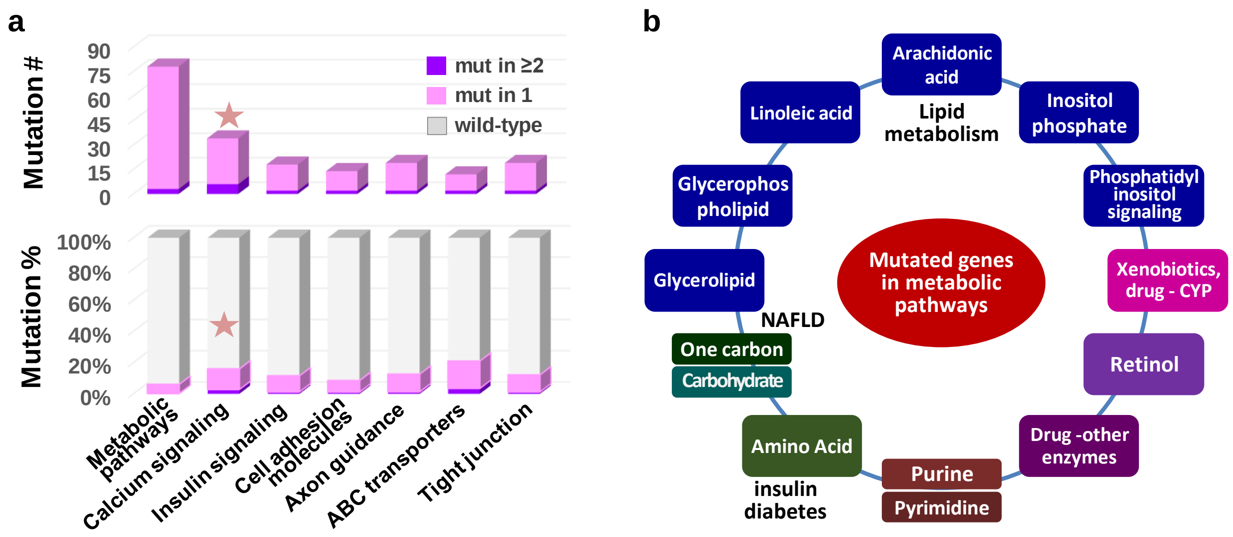

Supplementary Fig. 3 a Mutated gene numbers and proportions of affected pathways. **b** Forty-five of the 75 mutated genes in metabolic pathways are involved in specific metabolic pathways, 6 of which are associated with lipid metabolism.

Supplementary Fig. 4

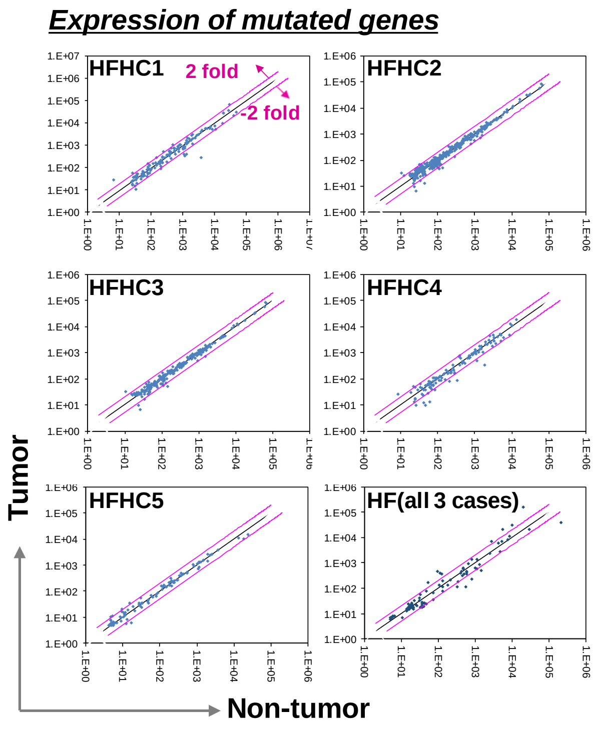

Supplementary Fig. 4 Dot plots showing the expression of mutated genes in HCCs as compared to adjacent non-tumorous livers.

Supplementary Fig. 5

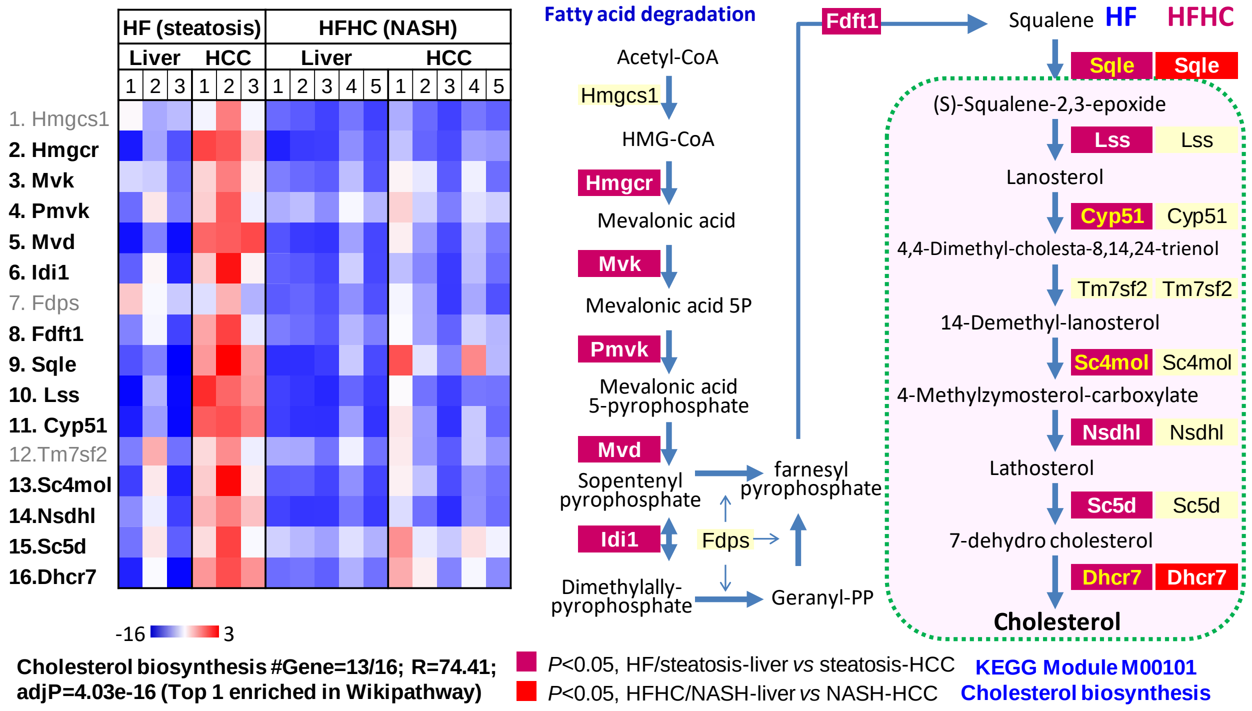

Supplementary Fig. 5 Genome-wide gene expressional analysis by microarray showed that 13 of the 16 genes in cholesterol biosynthesis pathway were significantly up-regulated in HF-induced steatosis-HCCs as compared with adjacent non-tumorous livers, while only two of these genes were up-regulated in HFHC-induced NASH-HCCs.

Supplementary Fig. 6

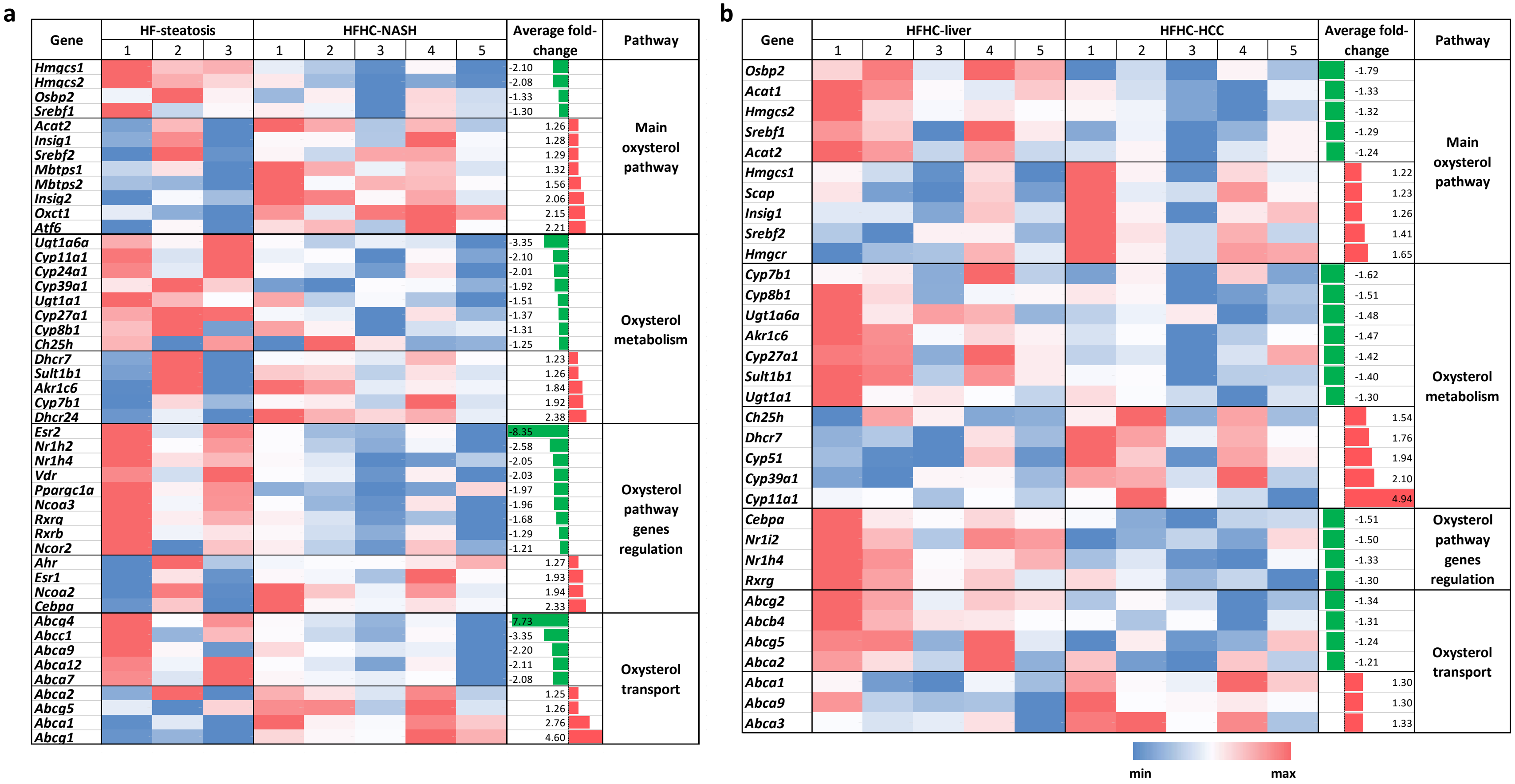

Supplementary Fig. 6. Expressional aberration of oxysterol pathway genes in NASH-livers compared to steatosis-livers (**a**), and in NASH-HCCs compared to adjacent non-tumor livers (**b**) in HFHC-fed mice. Expression levels were normalized to the mean level of each gene among all samples shown.

Supplementary Table 1. Mutated genes in calcium signaling

Sample	Gene#	adjP*	Mutated genes									
HFHC_87	7	0.0003	Ryr1	Ryr2	P2rx1	Nos2	Cacna1s	Itpr2	Itpr3			
HFHC_88	9	0.0004	Ryr1	Ryr2	Grin2c	Itpr1	Cacna1h	Cacna1d	Erbb3	Drd1a	Adcy8	
HFHC_89	10	1.10E-05	Ryr1	Ntsr1	Ptafr	Plcb2	Phka2	Cacna1d	Slc25a4	Atp2a3	Adcy4	Gna15
HFHC_93	5	0.0043	Ryr1	Plcg2	Htr7	Atp2a1	Cacna1h					
HFHC_96	5	0.0003	Plcd3	Pdgfra	P2rx1	Itpr1	Chrna7					
Recurrent	6	3.60E-06	Ryr1	Ryr2	P2rx1	Itpr1	Cacna1h	Cacna1d				

*adjP, p value adjusted by multiple test adjustment.

Supplementary Table 2. Mutated genes in metabolism-related pathways

#	PathwayName	Mutated genes (EntrezGene#)	Mutated Gene #	Gene # in pathway	adjusted P
1	Glycerolipid metabolism	110197 67800 50784 215456 102247	5	51	1.34E-08
2	Glycerophospholipid metabolism	110197 75320 50784 215456 102247 27388 665270	7	80	3.40E-11
3	Arachidonic acid metabolism	13096 107141 665270 404195 13098 71522 435802 13089 13090 631304 208285 11687	12	90	1.69E-20
4	Linoleic acid metabolism	13096 107141 665270 404195 13098 337924	6	46	7.44E-11
5	Phosphatidylinositol signaling system	72469 234779 18796 104015 110197	5	78	1.04E-07
6	Inositol phosphate metabolism	72469 234779 18796 104015	4	57	1.22E-06
7	Metabolism of xenobiotics by cytochrome P450	13096 337924 13089 404195 13090 107141 13098 94284	8	77	3.18E-13
8	Drug metabolism - cytochrome P450	13096 337924 13089 404195 13090 107141 13098 94284 213043	9	87	1.11E-14
9	Retinol metabolism	13096 337924 13089 404195 13090 107141 13098 94284 435802	9	77	4.72E-15
10	Drug metabolism - other enzymes	103149 337924 94284 382053	4	59	1.29E-06
11	Purine metabolism	18973 18971 18969 94041 231327 328099	6	168	1.40E-07
12	Pyrimidine metabolism	18973 18971 18969 103149	4	99	9.57E-06
13	Amino Acid metabolism	17448 18263 18948 15109 21990	5	112	7.92E-07
14	One carbon metabolism and related pathways	232087 108037 75320 246277	4	49	7.92E-07
15	Carbohydrate digestion and absorption	15277 226413 14377 18796	4	39	2.82E-07

Supplementary Table 3. Effective data and sequencing depth by whole-exome sequencing

Sample ID*	Effective data (Mb)	Mean depth	Coverage
HF_82T	5641.7	109.3	99.80%
HF_82N	5698.7	110.4	99.80%
HF_83T	5848.0	113.3	99.80%
HF_83N	5507.9	106.7	99.80%
HF_85T	5693.6	110.3	99.80%
HF_85N	5662.9	109.7	99.80%
HFHC_87T	6411.5	124.2	99.70%
HFHC_87N	5964.5	115.6	99.80%
HFHC_88T	5628.9	109.1	99.80%
HFHC_88N	5724.6	110.9	99.80%
HFHC_89T	5552.5	107.6	99.80%
HFHC_89N	5777.4	111.9	99.80%
HFHC_93T	5795.1	112.3	99.70%
HFHC_93N	5627.4	109.0	99.70%
HFHC_96T	5639.6	109.3	99.80%
HFHC_96N	5706.0	110.5	99.80%
**Mean**	**5742.5**	**111.3**	**99.78%**

*T, Tumor; N, adjacent non-tumor

Supplementary Table 4. Primers used in this study

Gene/target name	Primer	Nucleotide sequence (5'->3')	Size (bp)
CFD	Forward	CTTGATGTGCGCGGAGAG	139
	Reverse	GTAGATCCCGGGCTTCTTG	
DDIT3	Forward	AAGGCACTGAGCGTATCATGT	127
	Reverse	TGCTTTCAGGTGTGGTGATG	
ALDH18A1	Forward	TGTGGAGGGGAAGAAAGTTG	157
	Reverse	CAGATCAGCCAGATGATGGA	
PSPH	Forward	TCTTGCTCTTGCAGAATCCA	162
	Reverse	GCTCTGAGTGGGAGACCATC	
ITGA6	Forward	CGTGATCCGGAAATATGGAG	137
	Reverse	TGGCTCTCTGCAGTGGAAG	
CHKA	Forward	TCCAGTGCTCCCTACCTGAC	122
	Reverse	AGCTTGTTCGGATCCCTCTT	
SQLE	Forward	GCAAGCTTCCTTCCTCCTTC	103
	Reverse	CAACAGTCATTCCTCCACCA	
Beta-actin	Forward	GTCTTCCCCTCCATCGTG	113
	Reverse	AGGGTGAGGATGCCTCTCTT	
