# Supplementary material for: Dietary cholesterol promotes steatohepatitis related hepatocellular carcinoma through dysregulated metabolism and calcium signaling
Source: Nat Commun. 2018 Oct 26;9:4490. doi: 10.1038/s41467-018-06931-6 (PMC6203711; doi:10.1038/s41467-018-06931-6)
Supplement: Supplementary file 3 — Description of Additional Supplementary Files [file 41467_2018_6931_MOESM3_ESM.pdf]

Description of Additional Supplementary Files

File Name: Supplementary Data 1
Description: Somatic nonsynonymous mutations identified in liver tumors from high-fat (HF)-fed mice and high-fat-high-cholesterol (HFHC)-fed mice.

File Name: Supplementary Data 2
Description: List of recurrently mutated genes identified in liver tumors from HF-fed mice and HFHC-fed mice.

File Name: Supplementary Data 3
Description: Nine pathways significantly enriched by mutated genes in liver tumors from HFHC-fed mice

File Name: Supplementary Data 4
Description: List of mutated genes identified in liver tumors from HFHC-fed mice and verified in human NASH-HCCs.

File Name: Supplementary Data 5
Description: Genes involved in oxysterol pathways.
